# Oxygen Uptake Rate Soft-Sensing via Dynamic *k*_*L*_*a* Computation: Cell Volume and Metabolic Transition Prediction in Mammalian Bioprocesses

**DOI:** 10.3389/fbioe.2019.00195

**Published:** 2019-08-21

**Authors:** Magdalena Pappenreiter, Bernhard Sissolak, Wolfgang Sommeregger, Gerald Striedner

**Affiliations:** ^1^R&D - Bilfinger Industrietechnik Salzburg GmbH, Salzburg, Austria; ^2^Department of Biotechnology (DBT), University of Natural Resources and Life Sciences (BOKU), Vienna, Austria

**Keywords:** *k*_*L*_*a*, oxygen transfer rate, oxygen uptake rate, biomass prediction, metabolic states, quality by control, CHO

## Abstract

In aerobic cell cultivation processes, dissolved oxygen is a key process parameter, and an optimal oxygen supply has to be ensured for proper process performance. To achieve optimal growth and/or product formation, the rate of oxygen transfer has to be in right balance with the consumption by cells. In this study, a 15 L mammalian cell culture bioreactor was characterized with respect to *k*_*L*_*a* under varying process conditions. The resulting dynamic *k*_*L*_*a* description combined with functions for the calculation of oxygen concentrations under prevailing process conditions led to an easy-to-apply model, that allows real-time calculation of the oxygen uptake rate (OUR) throughout the bioprocess without off-gas analyzers. Subsequently, the established OUR soft-sensor was applied in a series of 13 CHO fed-batch cultivations. The OUR was found to be directly associated with the amount of viable biomass in the system, and deploying of cell volumes instead of cell counts led to higher correlations. A two-segment linear model predicted the viable biomass in the system sufficiently. The segmented model was necessary due to a metabolic transition in which the specific consumption of oxygen changed. The aspartate to glutamate ratio was identified as an indicator of this metabolic shift. The detection of such transitions is enabled by a combination of the presented dynamic OUR method with another state-of-the-art viable biomass soft-sensor. In conclusion, this hyphenated technique is a robust and powerful tool for advanced bioprocess monitoring and control based exclusively on bioreactor characteristics.

## Introduction

The primary role of a bioreactor is to provide a suitable environment for cell growth and product formation. Stirred tank reactors (STRs) are currently the most widely used bioreactor type to cultivate aerobic organisms in suspension culture or on carriers. In aerobic upstream bioprocesses, the oxygen uptake rate (OUR) is crucial for cellular activity and a good indicator of changes in the metabolic state of the culture (Deshpande and Heinzle, 2004; Wahrheit et al., 2015), which can be induced by changing substrate availabilities (Toye et al., 2010; Niklas et al., 2011; Young, 2013; Zhang et al., 2016). Thus, in the context of implementing Quality by Design and Process Analytical Technology (QbD/PAT) in bioprocesses, the OUR could be an informative process indicator (Sommeregger et al., 2017).

According to the QbD/PAT concept real-time measurements of meaningful process variables are a necessity. Soft(ware)-sensors can provide information about the actual state and quality of the process. Thereby on-line process variables are measured by associated sensors (hardware) using an estimation algorithm (software) to deliver estimated unmeasured bioprocess variables (Luttmann et al., 2012).

Before being consumed by cells, oxygen disperses through the culture medium in a series of transport resistances from gas bubbles to each individual cell. The highest resistance occurs during the transport through the liquid film surrounding the gas bubbles, which is described by the volumetric mass transfer coefficient (*k*_*L*_*a*). This coefficient and the concentration gradient ($c_{L}^{*}$-*c*_*O*2_) in the liquid phase defines the gas-liquid transfer rate and the oxygen transfer rate (OTR), respectively (Villadsen et al., 2011).

Precise OTR calculations during a bioprocess are challenging, because different phenomena occur simultaneously. Process conditions (e.g., pressure, temperature, mixing, and gas-flow) in a previously chosen operational mode (e.g., batch or fed-batch cultivation) together with physicochemical properties (e.g., media composition or viscosity) may change over time and influence the overall OTR (Garcia-ochoa and Gomez, 2009). Temperature and pressure greatly impact the maximum oxygen solubility in aqueous solutions, and therefore mainly influence the concentration gradient. Regarding the physicochemical properties of the medium, the amount of electrolytes (salts, ions) in so-called non-coalescing fluids can have beneficial effects on *k*_*L*_*a*, due to suppressing bubble coalescence (Villadsen et al., 2011). Other additives, such as Pluronic F68, which is typically added for shear protection, have been shown to reduce bubble size at high concentrations (Sieblist et al., 2013). Moreover, certain antifoam agents, such as silicone oils, can act as oxygen vectors, resulting in a significant increase in oxygen transfer and the oxygen transfer capacity in STRs (Quijano et al., 2009). In contrast, in bubble column reactors, *k*_*L*_*a* values decrease with the addition of hydrophilic or hydrophobic surface active compounds (Mcclure et al., 2015). In addition, increasing biomass particle size and by-product formation can reduce *k*_*L*_*a* values due to enhanced bubble coalescence (Vandu and Krishna, 2004).

In aerobic bioprocesses the dissolved oxygen concentration should not drop below a certain threshold. Therefore, a PID control circuit is usually used to counteract shortages. The output parameters of such a controller can be different among processes but usually includes stirrer speed, gas-flow or composition, pressure, or combinations thereof. By utilizing design of experiments (DoE), the influence of those parameters on *k*_*L*_*a* and $c_{L}^{*}$ can be determined within the operational process space. Consequently, OTR can be estimated at each time point during the process.

Though the OTR and *k*_*L*_*a* in particular are decisive parameters for the design of bioreactors, the OUR calculated in real-time provides information about the cells being cultured and the overall process performance. The OUR is a good indicator of cellular activity that closely correlates with the viable biomass. Within a bioprocess, the OUR is usually calculated via oxygen mass balancing. Therefore, the use of gas-analyzers is required to determine the oxygen and CO_2_ concentration in the off-gas stream, and these compounds can be quantified using flow rates. Another approach is to use the combination of OTR and the time-progression of the actual dissolved oxygen (DO) concentration (Lovrecz and Gray, 1994; Eyer et al., 1995). However, the published methods usually do not correct for changes in either *k*_*L*_*a* or $c_{L}^{*}$ due to process dynamics, or rely on empirical *k*_*L*_*a* calculations based on water experiments.

In this study, a soft-sensor was established for real-time estimation of the OTR and respectively, OUR. For this purpose, a 15 L bioreactor was thoroughly characterized to develop a dynamic model for *k*_*L*_*a* that can account for changing operational (temperature, PID controller output) and physicochemical properties of the medium (oxygen transfer and solubility). The model was applied, to a wide-spread dataset of 13 recombinant Chinese hamster ovary (CHO) cell culture fed-batch processes producing a monoclonal antibody (mAb) to elucidate the association of OUR with biomass and the metabolic states throughout the process. In summary, this study presents an estimation of the OUR based on standard measurements (PA and CO_2_ inlet gas flow-rates, temperature, volume, pressure) and precise system characterization that takes into account the dynamic *k*_*L*_*a* throughout progression of the process. This OUR soft-sensor was then used for biomass prediction. We also show an advanced technique for monitoring metabolic transitions of cells during cultivation simply by combining the dynamic OUR with a state of the art capacitance sensor.

## Materials and Methods

### Operational Conditions

A 15 L (max. working volume) stainless steel stirred tank bioreactor with a tank diameter (D) of 0.242 m and total height (H) of 0.484 m (LabQube, Bilfinger Industrietechnik Salzburg GmbH, Austria) was equipped with two three-bladed elephant ear impellers (d_i_ = 0.1 m) connected to a bottom-driven magnetic impeller shaft. Aeration was maintained by a submerged I-shaped frit and calibrated mass flow controllers (8711, Burkert, Germany). The temperature was measured using the built in Pt100 resistance thermometer. The DO concentration was monitored using an optical oxygen sensor (VisiFerm DO Arc120, Hamilton Switzerland) and pH by a pH probe (EasyFerm Plus PH Arc120, Hamilton, Switzerland). The oxygen and CO_2_ content in the off-gas stream was measured using a gas analyzer (BlueInOneFERM, BlueSens, Germany). A capacitance probe (Incyte, Hamilton, Switzerland) was used to evaluate the biomass estimations and establish the metabolism sensor.

### *k_*L*_a* Measurements

The experimental determination of *k*_*L*_*a* was performed using the dynamic gassing in/gassing out method (Van't Riet, 1979). Dissolved oxygen was measured by step changes in the oxygen concentration of the inlet gas. *k*_*L*_*a* was determined from the slope of the natural logarithmic DO concentration over time in an oxygen saturation range of 20–80%. Application of this method is restricted when the oxygen transfer is faster than the probe response. As proposed by (Van't Riet, 1979), the time constant of the measurement probe can be neglected if the following condition in Equation (1) is fulfilled:

(1) $$\begin{array}{r} \tau_{p}\leq \frac{1}{k_{L}a} \end{array}$$

As the mass transfer coefficients within the chosen process space for mammalian cell culture bio-production are relatively low, the response time determined for the used probe (τ_*p*_ = 49.6 s, experimentally) was sufficient. All measurements were performed according to a pre-defined experimental set-up with varying parameters (working volume, impeller speed, aeration rates, and culture temperature).

Two liquids, RO-H_2_O and a chemically defined culture medium (Dynamis AGT, A26175-01, Thermo Fisher Scientific, USA) both supplemented with 0.1% (v/v) antifoam C (A8011, Sigma Aldrich, Germany), were used to determine *k*_*L*_*a*. All measurements were performed in triplicate. Data accuracy was within ±5% for all measurements; thus, only the average values are shown in the respective depictions.

### Oxygen Transfer

Methods to quantify OUR and OTR are based on a gas-liquid mass balance of oxygen as described in Equation (2),

(2) $$\begin{array}{r} \frac{dC}{dt}=k_{L}a\;\left({c_{L}}^{*}-c_{O2}\right)-q_{O2}*X \end{array}$$

where the timely changes in oxygen concentration are influenced by the oxygen mass transfer coefficient (*k*_*L*_*a*), maximum solubility of oxygen ($c_{L}^{*}$), actual oxygen concentration (*c*_*O*2_), specific OUR (*q*_*O*__2_), and viable cell concentration (*X*). The OUR (*OUR* = $q_{O2}^{*}$X) and OTR are equal during steady-state conditions (controlled DO concentration), hence *dC/dt* = *0*, leaving OTR as described in Equation (3):

(3) $$\begin{array}{r} OTR=k_{L}a\;\left({c_{L}}^{*}-c_{O2}\right) \end{array}$$

The OUR model described in this work is based on a detailed bioreactor characterization, in which physiological and kinetic changes from a dynamic process, resulting in varying dynamic *k*_*L*_*a* values are considered. The on-line bioprocess data including the O_2_ and CO_2_ aeration rates, temperature, filling level and DO concentrations measured by an oxygen probe, as well as pre-determined oxygen solubility in water and cell culture medium were used for the model derivation.

### OUR Calculation by Oxygen Mass Balancing

One possibility for acquiring the consumed mass of oxygen on-line involves balancing the oxygen mass between the gas entering and leaving the bioreactor, which applies to animal cell cultures (Eyer et al., 1995). An accurate gas analyzer is required for this technique to measure the *Vol.O*_2, *out%*_ in the off-gas stream. In addition, the gas flow rate (*G*_*in*_= *G*_out_) and composition of the aeration gas that enters the bioreactor together with the liquid volume (*V*_*L*_) and molar gas volume [*V*_*m, in*_ (*p,T,R*); assumed *T*_*in*_= 22°C, *T*_*out*_= measured gas outlet temperature], needs to be taken into account to calculate the OUR as described in Equation (4):

(4) $$\begin{array}{r} OUR_{MB}=\frac{O_{2}\;in-O_{2}\;out}{V_{L}}=\;\frac{\left(\frac{Vol.O_{2in\%}*G_{in}}{V_{m,in}}\right)-\left(\frac{Vol.O_{2out\%}*G_{out}}{V_{m,out}}\right)}{V_{L}} \end{array}$$

### Maximum Oxygen Solubility: The Thermodynamic Approach

The maximum solubility of oxygen in water (c^*^) under ambient air was calculated using Equation (5), the temperature and pressure dependent thermodynamic equation described by Tromans (1998):

(5) $$c^{*}(T)=pO_{2}*\exp\left\{\frac{\begin{array}{l} 0.046\;T^{2}+203.357\;T\ln\left(\frac{T}{298}\right)\\ -\left(299.378+0.092\;T\right)\left(T-298\right)-20.591*10^{3} \end{array}}{R\;T}\right\}$$

*R* represents the ideal gas constant and *T* the temperature in K.

### Determination of Oxygen Solubility in Medium

To investigate the solubility of oxygen in the presence of (non)ionic compounds and sugars, the solubility in the cell culture medium was determined experimentally as described by (Storhas, 2018). Briefly, in two steps, either oxygen saturated or degassed RO-H_2_O with known Henry coefficient was mixed in equal amounts with the cell culture medium and the resulting dissolved oxygen concentration was measured (DO_1_). The second value (DO_2_) is determined using the same liquids with vice versa oxygen saturations. The obtained difference was used to correct the maximum absolute oxygen saturation in media.

### CO_2_ Influence on Solubility in Medium

Changing CO_2_ concentrations in the gas inlet due to pH control influence the oxygen solubility in the culture. To analyze the maximum saturation in the presence of CO_2_, gassing experiments applying up to 20% (v/v) CO_2_ in process air were performed and the maximum oxygen solubility in cell culture medium recorded.

### Fed-Batch Experiments

#### Cell Line Propagation

We used a recombinant monoclonal Chinese Hamster Ovary (CHO) cell line (Antibody Lab GmbH, Austria) generated by the *Rosa26* Bacterial Artificial Chromosome (BAC) strategy (Zboray et al., 2015) using a serum-free derivate of CHO-K1 (ATCC CLL-61) as the host. The cells produce an IgG1 monoclonal antibody. The cell line was cultured in chemically defined cell culture medium (Dynamis AGT, A26175-01, Thermo Fisher Scientific, USA) supplemented with 8 mM L-glutamine. The cells were maintained in shake flask cultures at 37°C in a humidified incubator under 5% (v/v) CO_2_ in air, shaken at constant rpm and passaged every 3–4 days for propagation and scale-up. After four passages, the bioreactor was seeded at a density of 2.5 × 10^5^ cells mL^−1^.

#### Bioprocess

Thirteen CHO cultivations were performed in a chosen (DoE) setting with either static or dynamic changes (intra-experimental shifts) in two varying parameters. The changeable parameters were temperature and variable D-glucose concentration in the feed medium (see Table 1). For simplification, runs 1 and 2 were treated as processes performed at 37°C.

**Table 1:** Overview of all bioprocess runs at different parameter settings.

**Bioprocess run**	**Shift 1** **(72 h)**	**Shift 2** **(120 h)**	**Shift 3** **(192 h)**	**Shift 4** **(240 h)**
1	36.3°C/F3	–	–	–
2	36.3°C/F3	–	–	–
3	34°C/F1	–	–	–
4	37°C/F3	-	37°C/F1	-
5	34°C/F2	37°C/F2	34°C/F1	31°C/F1
6	31°C/F2	34°C/F2	37°C/F3	34°C/F3
7	34°C/F1	31°C/F1	31°C/F2	34°C/F2
8	37°C/F2	34°C/F3	31°C/F2	34°C/F1
9	34°C/F3	37°C/F2	31°C/F2	37°C/F3
10	34°C/F2	–	–	–
11	34°C/F2	–	–	–
12	34°C/F2	–	–	–
13	34°C/F2	–	–	–

*Intra-experimental variations were performed at four shifts in temperature (31, 34, or 37°C), additional D-glucose concentration in the feed medium (+10, +20, or +30 g L^−1^; identified as F1, F2, F3), or both*.

The feed medium (CHO CD EfficientFeed A, A1442001, Thermo Fisher Scientific, USA) was supplemented with 0.1% (v/v) antifoam C (A8011, Sigma Aldrich, Germany) and additional 10, 20, or 30 g L^−1^ D-glucose and 7 g L^−1^ L-asparagine monohydrate. Temperature was maintained at 37°C during the batch phase and changed after 72 h to 31 or 34°C or remained constant according to the pre-defined experimental set-up. The minimum DO level was set to 30% of saturation and maintained by gassing with process air (PA) flow and increasing stirrer speed. The agitation rate was variable, from 91 to 228 rpm and the gas-flow range was 0.3–1.5 L min^−1^ (maximum values at maximum PID controller output). The culture pH was kept constant at 7.0 and controlled via the CO_2_ gas flowrate. Base addition was not necessary.

### Off-line Analyses

The total cell concentration (TCC) was determined by counting the cell nuclei using the particle counter Z2 (Beckman Coulter, USA). Therefore, an appropriate amount of cell suspension was centrifuged at 200 *g* for 10 min. The cell pellet was resuspended in 0.1 M citric acid monohydrate and 2% (v/v) Triton X-100 buffer to lyse the cells, for a minimum of 1 h before measurement. Sample dilution was performed using a 0.9% (m/m) NaCl solution.

Culture viability was assessed using a hemocytometer and trypan blue exclusion. The viable cell concentration (VCC) was calculated by multiplying viability by TCC.

Packed cell volume (PCV) was measured using PCV tubes (#87007, TPP, Switzerland) after spinning the cell suspension for 1 min at 2,000 *g*. PCV is expressed as a percentage (%v/v) of the total culture volume. Determinations were performed in duplicates. Viable PCV was determined by multiplying the viability by the PCV.

Carbohydrates were determined via ion exclusion chromatography (HPX 87H, 300 × 7.8 mm, #1250140, BioRad, USA) on an Agilent 1200 series (Agilent, USA) at 25°C. The mobile phase consisted of 5 mM sulfuric and the flow rate was set to 0.45 mL min^−1^. Measurement was performed using a Refractive Index detector (35°C). The calibration range for D(+)-glucose was 100–2,000 mg L^−1^. The chromatograms were evaluated using Chemstation software (revision B.04.01, Agilent, USA).

The amino acid concentrations were determined by HPLC. After using an automated pre-column derivatization method, amino acids were separated on a chromatography column (Eclipse Plus C18 column) at 40°C using a flow rate of 0.64 mL min^−1^. As solvent A 10 mM K_2_HPO_4_ and 10 mM K_2_B_4_O_7_ and Solvent B an acetonitrile, methanol, water mix (45/45/10; %v/v/v) was used. Amino acids were excited at 230 nm and the fluorescence signal was detected at 450 nm for OPA derivatives and at 266/305 nm for FMOC derivatives. Samples were quantified using an internal standard calibration.

### Assessing Model Accuracy

To compare the model's quality, accuracy, and overall performance, the mean absolute percentage error (MAPE) was calculated. Errors were normalized by the inverse of the number of fitted points (*n*) regarding the sum of deviation from actual values (x_i_) to forecast values (x_target_) divided by the actual value again, calculated as a percentage error (%) as described in Equation (6):

(6) $$\begin{array}{r} MAPE=\frac{\sum_{i=1}^{n}|\frac{x_{i}-x_{target}}{x_{i}}|}{n}*100 \end{array}$$

## Results

### Assessing Parameters for Dynamic *k_*L*_a* Estimation

As oxygen transfer is determined by the system's operational and physicochemical characteristics, varying process conditions can affect the oxygen solubility and mass transfer properties and need to be taken into account for *k*_*L*_*a* model development.

**Figure 1:**
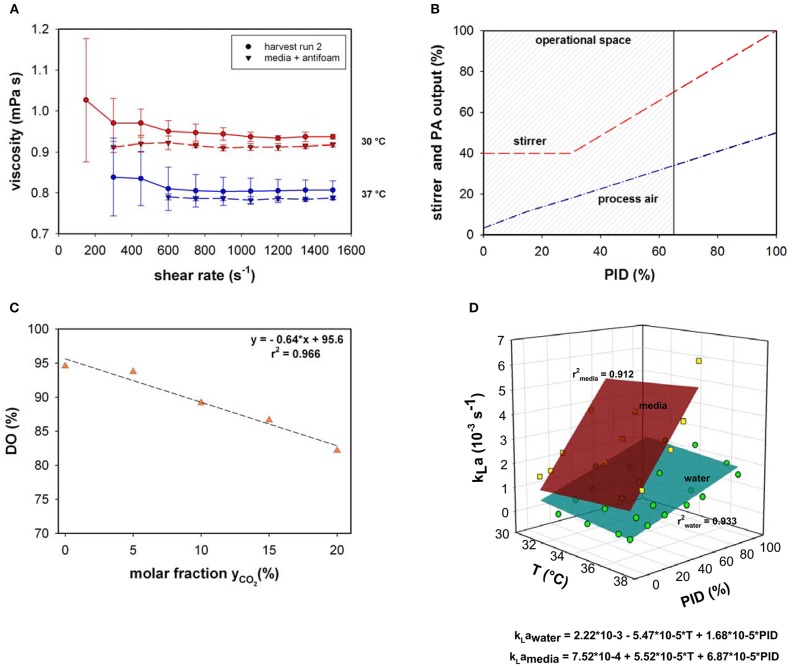
**(A)** Viscosity as a function of shear rate measured in cell suspension and media supplemented with antifoam at 30 and 37°C. **(B)** Stirrer speed and process air (PA) output as a function of PID controller output (%). **(C)** Relative dissolved oxygen saturation (DO%) determined in medium upon variation of the molar CO_2_ fractions in the inlet gas compared to RO-H_2_O. **(D)** *k*_*L*_*a* as a function of temperature (*T*) and PID controller output (%) for water and culture medium supplemented with 0.1% v/v antifoam solution.

Viscosity behavior was investigated using the harvest samples of bioprocess run 2 and media supplemented with antifoam at two different temperatures (30 and 37°C; Figure 1A). The viscosity was close to that of water and significant changes between the media and harvest sample were not observed. Due to the insignificance of the divergence, viscosity changes were not implemented in the present model.

Osmolality within all presented fed-batch processes was 295 ± 26 mOsm kg^−1^. Similar to the viscosity, the minor osmolality variations were assumed to only minimally influence the maximum oxygen solubility in culture medium or the *k*_*L*_*a* and therefore, were neglected.

Volumetric mass transfer coefficients were measured in a chosen process design space (Figure 1B). Process air and stirrer speed variation were linked in the PID controller output; therefore, the influence on *k*_*L*_*a* was quantified based on the percentage of the PID controller output. During all fermentations, the main operational space increased to a maximum PID controller output of 65%. At maximum operational stirrer speed (PID65 = 160 rpm), a specific agitation power of 12 W m^−3^ was calculated.

The volume dependency of *k*_*L*_*a* between 10 L (inoculum) and 15 L (max. working volume) was investigated experimentally. No significant volume influence on *k*_*L*_*a* was determined in the bioreactor system.

As the pH in mammalian cell culture processes is typically controlled by varying the CO_2_ concentration in the inlet gas, the maximum oxygen solubility in cell culture medium was determined under varying CO_2_ molar fractions in the gas in-flow (Figure 1C). Gassing with ambient air led to a maximum relative solubility of ~95 % in media compared to water under the same settings. This result is in accordance with the experimentally determined maximum oxygen solubility in medium compared to water using the method described by (Storhas, 2018), resulting in a decrease of 5.2% in culture medium compared to RO-H_2_O. With increasing molar fractions of CO_2_ the oxygen solubility dropped to 82% at a molar CO_2_ fraction of 20%. The resulting linear fit was incorporated into the model to account for O_2_ displacement by CO_2_ (Equation 8).

The physicochemical properties of the culture medium had a strong positive impact on *k*_*L*_*a* values in this bioreactor setup (Figure 1D). The *k*_*L*_*a* values determined with medium were more than 3-times higher than those generated with water in the presence of 0.1% v/v antifoam solution. A linear increase was observed in *k*_*L*_*a* in cell culture medium with increasing PID (PID = f(*v*_*s*_*,rpm*). The increase in superficial gas velocity together with increasing stirrer speed as a function of PID output had the greatest impact, whereas temperature had only a slight effect. A linear curve fit was created with averaged triplicate values. The *k*_*L*_*a* determination in medium was performed up to a PID controller output of 60%, with linear extrapolation of higher values. This function was used to estimate *k*_*L*_*a* in real-time throughout the process as the PID set-up was the same for all fed-batches. By determining factors that directly influence oxygen solubility, several correlations have been developed for the prediction of *k*_*L*_*a* (Gill et al., 2008; Garcia-ochoa and Gomez, 2009). The most common and conventional approach is based on the energy input criterion. However, direct relation of *k*_*L*_*a* dependence to volumetric power consumption (*P*_*g*_*/V*_*L*_) or superficial gas velocity (*v*_*s*_) was not necessary due to coupling via the PID controller. The simplified model *k*_*L*_*a* = *f(T, PID)* is only true within the chosen process space and needs to be adapted for prevalent use. As an alternative, computational fluid dynamics (CFD) simulations can provide a tool for predicting *k*_*L*_*a* on larger scales in which the location of the oxygen probe in the bioreactor plays a significant role (Kerdouss et al., 2008; Wutz et al., 2016).

### OUR Model Set-Up

OUR at time point *t* is a function of the dynamic *k*_*L*_*a* and the oxygen solubility at given temperature c^*^(T) as described in Equation (7) (adapted from Equation 2).

(7) $$\begin{array}{r} OUR\left(t\right)={k_{L}a}_{dyn.}\left(t\right)*\left(c_{M}^{*}\left(t\right)-c_{DO}\left(t\right)\right)-\frac{dC}{dt} \end{array}$$

Dissolved oxygen concentrations with the subscript DO were obtained from the DO values measured by the oxygen probe. The *dC/dt* term equals zero if DO is constant. At the beginning of the processes, when DO was not constant, *dC/dt* in the short interval of on-line recording (seconds) was much smaller than OUR. Therefore, *dC/dt* was neglected for the on-line OUR calculations described in this work.

Oxygen solubility in fermentation medium $c_{M}^{*}$(t_1_*)* was calculated using the thermodynamic equation in the presence of medium solutes (Equation 8) and accounts for the O_2_ displacement by CO_2_.

(8) $$\begin{array}{r} c_{M}^{*}\left(t_{1}\right)=\;c^{*}\left(T\right)\left(\frac{-0.638^{*}y_{CO_{2}}\left(t_{1}\right)+95.63}{100}\right) \end{array}$$

with

(9) $$\begin{array}{r} y_{CO_{2}}=\frac{Q_{CO_{2}}}{\left(Q_{PA}+Q_{CO_{2}}\right)}*100 \end{array}$$

Q represents the inlet gas flowrate of CO_2_ (*Q*_*CO*2_) and process air (*Q*_*PA*_) gathered from the mass flow controller. Therefore, $c_{M}^{*}$(t_1_*)* is dependent on the process temperature and amount of dissolved CO_2_.

*DO(t*_1_*)* is the dissolved oxygen measured at the respective time point. In addition, as the used DO probe performs an internal temperature correction, a correction factor was introduced for the temperature dependence of the actual oxygen saturation. *c*_*DO*_*(t1)* is then defined as described in Equation (10):

(10) $$\begin{array}{r} c_{DO}\left(t_{1}\right)=\;c_{M}^{*}\left(t_{1}\right)*\left(DO\left(t_{1}\right)*\frac{c^{*}\left(T_{1}\right)}{c^{*}\left(T_{2}\right)}\right) \end{array}$$

### Assessing the Model Performance

**Figure 2:**
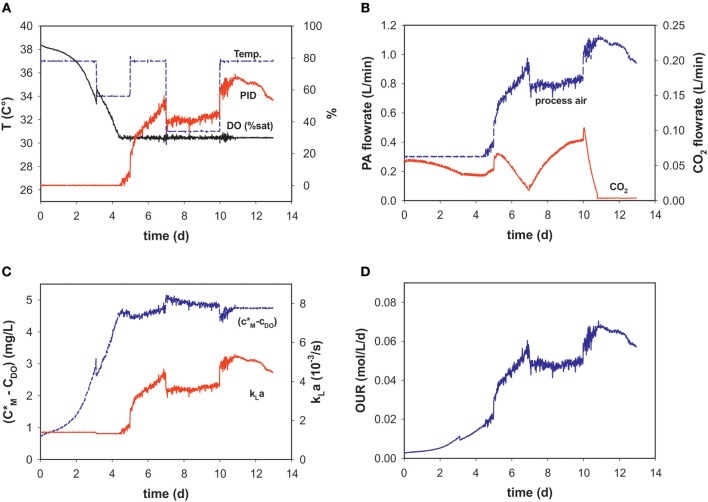
Process variables **(A)** temperature (*T)*, dissolved oxygen (DO% sat), and PID output (%), **(B)** process air (PA) and CO_2_ flow rates, **(C)** calculated solubility difference (c^*^_M_*-c*_*DO*_), *k*_*L*_*a* and **(D)** OUR over the time course of an intensified fed-batch process (run 9).

During the dynamic fed-batch processes (see Table 1) up to four temperature shifts were applied to capture the process dynamics (Figure 2A). Process air-flow at the beginning of the process was usually set to 0.3 L min^−1^ to constantly strip CO_2_, and increased with cell density when the set point of 30% DO was reached (Figures 2A,B).

Temperature-shifts influenced the PID output, as a reason of the temperature dependency of the maximum oxygen solubility. It follows that the temperature shifts are also evident in the concentration gradient (Figures 2A,C). With changing PID output and temperature, *k*_*L*_*a* changes over the progression of the fed-batch process. These dynamic profiles are very similar to those of the PID and PA-flow, as the main factor influencing *k*_*L*_*a* within the chosen process space is the superficial gas velocity (Figures 2B,C). After temperature correction and incorporating oxygen solubility, the OUR profile is calculated in real-time (Figure 2D).

**Figure 3:**
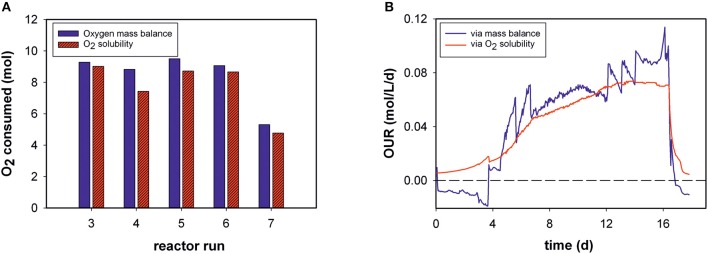
**(A)** Total oxygen consumption determined by the two different methods for five bioprocesses **(B)** Volumetric oxygen uptake rates (OURs) over the time course of fed-batch run 3. For clarity, only every 20th data point is displayed.

Total amounts of mol O_2_ consumed during each process were determined for five bioprocesses (Figure 3A). The results obtained with the generated model and values calculated by the mass-balance method were in good agreement (Equation 4). The O_2_ solubility approach in medium compared to the mass-balance method for all runs obtained slightly lower values. The mean relative deviation of the model compared to off-gas analysis was 8%. Due to humidity in the off-gas stream as well as handling errors, not all reactor runs could be evaluated by mass balance.

For example, Figure 3B shows the calculated OUR trend of a static fed-batch (run 3) in the developed model and the mass-balance method over the duration of cultivation. The same trends were gathered from both methods. However, the generated model seems to be unaffected by process disturbances. In particular, the rate calculation at the beginning of the processes, was mostly negative for the gas balancing method, whereas the soft-sensor illustrates the initial process phase in an exponential increase. Step changes and fluctuations during the process (especially temperature shifts) also impacted other reactor runs for the mass-balance method.

### Linking OUR to Cell Numbers

**Figure 4:**
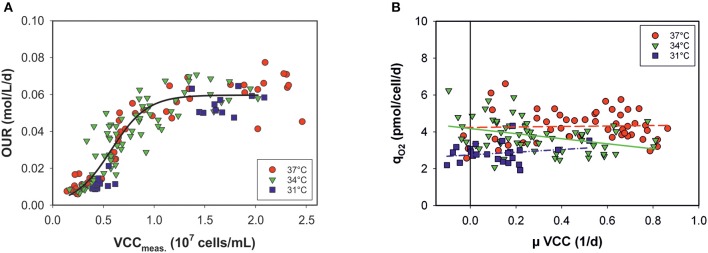
**(A)** Volumetric oxygen uptake rates (OURs) as a function of viable cell concentrations (VCCs) measured off-line for all bioprocesses. Data <80% viability were excluded. **(B)** Specific consumed oxygen rates (*q*_*O*2_) as a function of the growth rate (μ) for three culture temperatures (31, 34, and 37°C).

In principle, estimation of the OUR provides broad information on cell performance during the process. The OUR is the direct product of specific consumed oxygen rates (*q*_*O*2_) and the number of viable metabolically active cells. Thus, the OURs calculated by the model can be theoretically given as a function of VCCs measured off-line at each time point (Figure 4A). However, the OUR was linearly dependent only up to a cell concentration of ~10^7^ cells mL^−1^. At higher cell densities and later process stages, variations occur and the data are widely scattered: OURs no longer exhibit a clear relationship with the VCC. The data distribution indicates two process stages in the cells. Volumetric oxygen uptake is temperature-independent in a sigmoidal progression of cell numbers. The cell-specific oxygen consumption rate (*q*_*O*2_) is independent on the growth rate (μ) and the cell cycle, with constant, but temperature-dependent behavior (Figure 4B). Linear regression was carried out for each culture temperature.

### Linking OUR to Cell Volume

**Figure 5:**
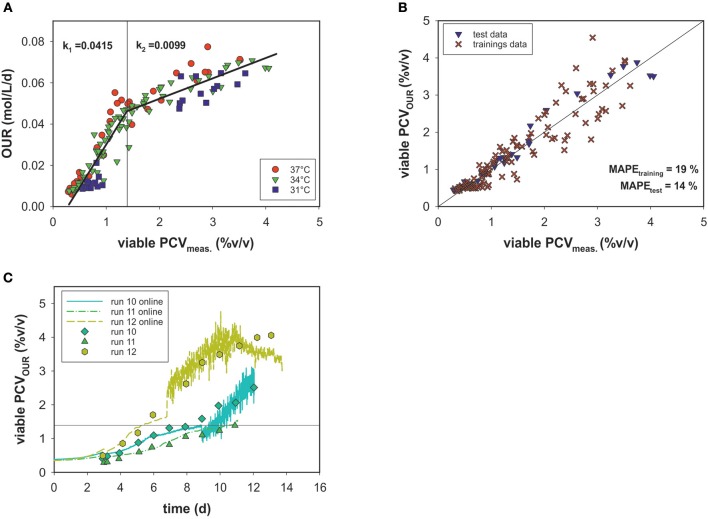
**(A)** Linear correlation of OUR and viable PCV for three culture temperatures (31, 34, and 37°C). Data with <80% viability were excluded. **(B)** PCV estimated by the segmented linear model vs. viable off-line PCV data. **(C)** PCV model predictions over time for the test data compared to values measured off-line.

An alternative means for biomass quantification in cell culture processes is the determination of PCV, which represents the average cell volume and closely correlates with oxygen uptake (Wagner et al., 2012). A growth profile comparison of cell numbers and PCV showed different curve characteristics (see Appendix Figure 1). Due to intra-experimental shifts in two parameters (added D-glucose in the feed medium and culture temperature), the viable PCV data as a function of time in all fermentation runs spanned a broad range. Nevertheless, these variations are not visible when correlating the viable cell volumes measured off-line to the OURs of all runs (Figure 5A).

Figure 5A shows that the magnitude of the OUR was highly dependent on PCV. No significant temperature dependence or association to the cell viability (>80%) was evident. The data are less scattered and a more accurate correlation, in comparison to cell number is observed. However, the OUR as a function of PCV exhibited a sharp kink at ~1.4% (v/v) PCV. Thus, a linear regression was calculated for each section. For this purpose, the data were divided into a training data set and a test data set. The test set consisted of three similar experiments with static conditions (runs 10, 11, and 12). All other experiments were used for development of the model. The optimal point of intersection between the two linear fits was calculated iteratively at 1.395% (v/v) PCV. For the first section, a linear function of *f*_1_= 0.042^*^x−0.011 and for the second one *f*_2_ = 0.001^*^x + 0.032 was calculated, where the slope *k* represents the specific uptake rate per cell volume in the respective section. After the transition, the OUR decreased and *k*_2_ was roughly one-fourth the value of *k*_1_. The error for the biomass prediction was calculated as MAPE = 19% for the training data set and 14% for the test data set (Figure 5B). Good performance of the predicted PCV was also seen in comparisons with the real PCV data for the test data sets (Figure 5C) over the time course of the process. Interestingly, two growth curves (runs 12 and 13) were still during exponential growth phase when reaching a PCV of ~1.4% (v/v). Therefore, partitioning the data into two stages could not be linked to the cell cycle (growth and maintenance).

### Capacitance Measurements for Soft-Sensing of Cell Volumes

**Figure 6:**
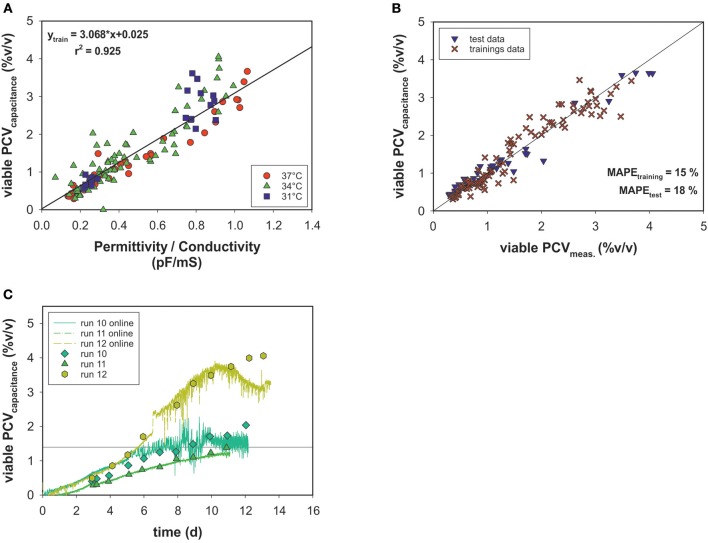
**(A)** Linear model of on-line capacitance signals as a function of viable PCV off-line data. **(B)** Estimated vs. real off-line PCV. **(C)** PCV capacitance model predictions over time for the test data.

Off-line PCV data correlated with the permittivity and conductivity signals of an on-line capacitance probe (Figure 6A). The permittivity exhibited linear behavior relative to the viable PCV, and conductivity output was used to correct the model for temperature changes. The cell factor determined by linear regression was used to predict the viable PCV. Again, data were split into a training data set and a test data set and the model's performance was evaluated using MAPE_training_ = 15% and MAPE_test_ = 18% for the on-line biomass soft-sensor (Figure 6B). The on-line soft sensor estimated the PCV trends for the test data sets over time in a meaningful manner (Figure 6C).

### Monitoring Metabolic Transitions

The transition step in the OUR profile indicated that a metabolic shift occurred (see Figure 5A) at a viable PCV of 1.4% (v/v) in the given process set-up.

**Figure 7:**
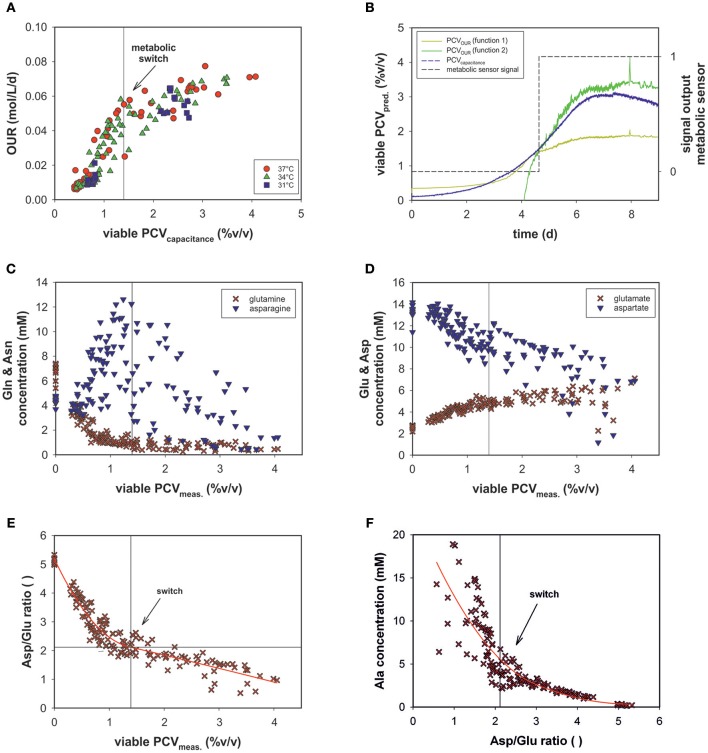
**(A)** OUR combined with PCV prediction by on-line capacitance probe signal of all bioprocesses. **(B)** The performance of the metabolic sensor in run 12 predicts the metabolic shift occurring in the culture. **(C)** Metabolic profiles of all fed-batch runs of glutamine (Gln) and asparagine (Asn) **(D)** glutamate (Glu) and aspartate (Asp) as a function of viable PCV. **(E)** The ratio of aspartate/glutamate (Asp/Glu) vs. viable PCV. **(F)** Alanine concentration (Ala) vs. the Asp/Glu ratio.

The evolved OUR model combined with the viable PCV predictions via on-line permittivity and conductivity signals from a capacitance sensor of all fed-batch runs is shown (Figure 7A). The combination of the two models led to the development of an on-line metabolic soft-sensor (see Appendix Figure 3). Hereby, the objective function is that the linear function PCV_OUR_ (function 1) must intercept with PCV_OUR_ (function 2) and the PCV_capacitance_. The sensor specifies the first metabolic state with the value 0 and the second with the value 1. If both conditions are fulfilled, hence, a metabolic shift is evident, the sensor jumps from 0 to 1. For 12 runs the application of the metabolic sensor was successful. In average, the metabolic shift was detected at a viable PCV of ~1.4% (v/v). As an example, the performance of run 12 is depicted in Figure 7B. During the process the metabolic shift can be traced by the output signal of the metabolic sensor.

The variations in the amino acid concentrations of glutamine (Gln), glutamate (Glu), asparagine (Asn), aspartate (Asp), and alanine (Ala) are of particular importance in mammalian cell culture (Zhang et al., 2016). Though Glu and Gln exhibited a similar trend over the course of the fed-batch for all experimental runs, Ala, Asn, and Asp progressed differently (see Appendix Figure 2).

In a recent study, the ratio of asparagine and glutamine was found to be important, to some extent, in terms of process performance (Zhang et al., 2016). However, glutamine was depleted and asparagine concentrations were sufficiently high throughout the process (Figure 7C) and did not exhibit significant dependence. In the metabolic fate of glutamine and asparagine, glutamate and aspartate, respectively follow as secondary key products (Figure 7D). The Asp concentration decreased, whereas the Glu concentration increased consistently. These amino acids exhibited a linear relationship with OUR (data not shown).

Glu and Gln as a function of PCV exhibited reciprocal behavior. At ~1.4% (v/v) PCV, glutamine was almost completely consumed and glutamate plateaued at ~6 mM (Figures 7C,D).

The Asp/Glu ratio as a function of PCV exhibited a significant pattern (Figure 7E). The Asp/Glu ratio decreased linearly. At a PCV value of ~1.4% (v/v), the progression bent and resulted in a shallower slope. Accordingly, at an Asp/Glu ratio of 2, cell volumes and specific OURs changed after a metabolic alteration. The observed inflection point was at the same value as in the OUR vs. PCV regression (1.4% (v/v) PCV; see Figure 5A).

The Ala profile suggested the same metabolism switch (Figure 7F). The Ala concentration slightly increased at high Asp/Glu ratios until a certain point (around 2), when Ala production started to increase steeply (Asp/Glu = low). Ala accumulated in the cell culture supernatant to a great extent.

## Discussion

### Benefit of Dynamic *k_*L*_a* Determination and Real-Time OUR Calculation

The dynamic technique for *k*_*L*_*a* determination provided reliable results. We showed that the assumption of a dynamic volumetric mass transfer coefficient is necessary to calculate the OTR and, subsequently, the OUR throughout a changing process. Temperature and PID controller output were the two main bioreactor operating variables affecting the OTR in this setting. The influence of physicochemical properties of certain substances in the cell culture medium led to a strong *k*_*L*_*a* increase more than 3-times higher compared to water. This is probably due to the presence of Pluronic F68 within the medium, which has been reported to mainly change bubble size at higher concentrations. Smaller bubbles lead to an increase in gas holdup and available surface areas for overall mass transfer (Sieblist et al., 2013). Similar results were reported in the presence of ionic solutes, which generally exhibit coalescence-inhibiting characteristics, resulting in smaller bubbles and greater surface area and overall *k*_*L*_*a* (Puthli et al., 2005). Moreover, the effects of so-called oxygen vectors (e.g., hydrocarbons, oil as antifoam agents) can enhance mass transfer rates to significantly higher levels than in water. The enhancement was mainly due to an increase in the air/water transfer rate, which is partially explained by the change in the water surface tension (Morao et al., 1999; Quijano et al., 2009).

According to these observations, the influence of culture medium composition on oxygen mass transfer has to be considered. Moreover, a maximum decrease was recorded in the relative oxygen saturation of ~18% in fed-batch medium during CO_2_ stripping. These results demand particular consideration of solubility changes with shifts in gas composition and temperature. Considering only the saturation O_2_ concentration in water instead of determining the prevailing saturation concentrations would lead to inaccuracies during specific OUR calculations (Henzler and Kauling, 1993).

The application of the dynamic OTR as a soft-sensor for calculating the OUR is demonstrated by the highly linear relationship between OUR calculated by a global mass-balance and OUR calculated by the model for a wide range. The presented model enables real-time prediction of the OUR without sophisticated off-gas measurements. The advantage of this approach is that it is simply based on DO measurement, knowledge of oxygen solubility properties in the medium, and recording process temperatures, pressure and volumetric inlet flow- rates of PA and CO_2_. The established model is in good agreement with the conventional technique. The minor off-set due to the inlet gas flow temperature for the mass-balance method was not determined, as it was generally assumed to be 22°C. More importantly, the generated profiles were smooth and, even at temperature shifts, no great disturbances were observed. Due to fluctuations from the off-gas analyzer, the methods could not be compared for every process run.

Overall, the established model with incorporated dynamic *k*_*L*_*a* determination demonstrated high potential for online monitoring of (specific) OURs during a cell culture bioprocess. This concept can be realized for all aerobic bioprocesses. However, in the field of microbial fermentation, where the *k*_*L*_*a* values can be up to more than 10-times higher, the probe response time used for *k*_*L*_*a* determination needs to be considered.

Moreover, the developed method has high potential in parallel bioreactor systems since it only relies on physical parameters. Therefore, once one bioreactor is characterized, the model may be transferred to all equivalent ones. However, in small-scale systems the experimental design may need to be adapted due to diverging influence factors arising from difference in scales or media.

### Application of Dynamic OUR for Cell Monitoring

In principle, an estimation of the OURs provides broad information on the overall process performance during the process but does not report detailed information about cell growth and physiology. The OUR is the direct product of the specific consumed oxygen rate *q*_*O*2_ and the biomass (Wagner et al., 2012). Conclusively, the prediction of biomass via oxygen consumption should be possible if *q*_*O*2_ was constant over the process progression.

Temperature changes impact cell growth and size, and this applies to respiration as well (Moore et al., 1997; Goudar et al., 2011). By presenting the OUR as a function of viable cell concentration, no clear temperature dependency was observed (see Figure 4A). Data from all bioprocesses were equally distributed in a sigmoidal progression. At later process stages no linear behavior was observed between the OUR and VCC. After a switch in the cellular metabolism, OURs seem to approach a plateau, independently of increasing cell concentrations. However, the cell physiology changed during the progression, affecting the OUR but this was not accounted for by the model. A minor temperature influence on *q*_*O*2_ was observed when plotted against the growth rate, μ, of the viable cells. Cells seem to require less oxygen at lower temperatures. Nevertheless, a dependency on cell cycle and growth was ruled out.

**Figure 8:**
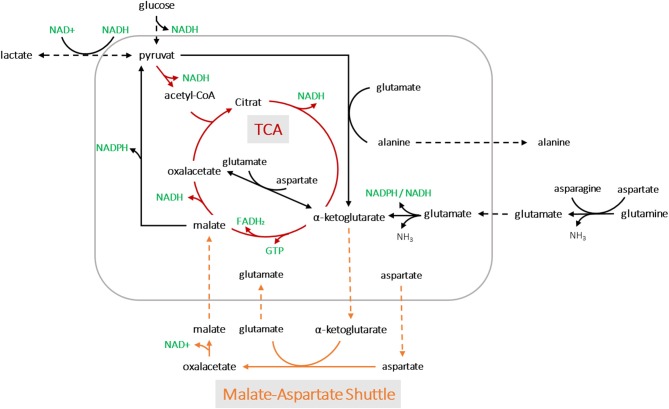
A simplified metabolic network of CHO-K1 cells. The detection of two metabolic states led to possible pathway assumptions in the TCA cycle and amino acid metabolisms for product synthesis.

Applying the model for accurately predicting viable cell number has its limitations, especially during later process stages, most likely due to changes in the cell size. In this study, we showed that oxygen consumption is rather related to cell volume than to cell count. Another study has also pointed to this fact (Wagner et al., 2012). A segmented linear model was established, able to cope with the metabolic shift occuring during the process. Remarkably, the clear metabolic shift was evident for all process runs despite massive variations within the design space and the segmented linear model could cope with it. The prediction error was calculated using a MAPE_training_ of 19% and MAPE_test_ of 14%, but due to the shallower slope in the second segment, the PCV prediction was more prone to error at higher cell volumes. This can be explained by the fact that cell growth includes an increase in both cell volume and number. Thus, deviations occur, particularly in stationary and death phases, when cell lysis is followed by the presence of cell fragments and increased aggregates (Lovrecz and Gray, 1994). The shift in the metabolic state of the cells led to roughly a quarter less oxygen consumption in the second stage, which may be driven by a truncated TCA cycle (Figure 8). Glutamine and other amino acids can have alternative fates entering the citrate cycle to supply ATP and/or NADPH. In a truncated cycle, less energy is produced and less oxygen is consumed. The OUR soft-sensor allows the viable cell volume to be predicted with reasonable accuracy. The method represents a simple and economic solution for bioprocess monitoring as no additional (off-gas) sensor systems are required.

### On-line Soft-Sensing of Cell Volumes Using a Capacitance Probe

The on-line monitored permittivity signal during each bioprocess was converted directly into a more meaningful dimension, the viable PCV, using the correlation (cell) factor predetermined with a linear regression. As a result, a temperature-independent function was generated using the conductivity signal for temperature correction, but no metabolic transition was observed. The estimated vs. measured values exhibit a normal distribution and, with respect to accuracy, all states could be determined with an adequate MAPE which is comparable to deviations in the two-segment linear model predictions (Figure 6B). The trends for the test data set were calculated with acceptable estimated errors: a drift in the estimation was observed only in the stationary and death phases. The time-resolved information obtained by the soft-sensor could be linked to the OUR soft-sensor for real-time identification of metabolic behavior in mammalian cell culture processes.

### OUR as a Metabolic Sensor

We assumed that varying process conditions (e.g., altered temperature profiles and D-glucose concentrations) during all bioprocesses may trigger different cellular responses with respect to oxygen consumption. However, all cells tended to alter their metabolic activity to a different state at a certain point, regardless of whether they were cultured in a dynamic or static process. In all fed-batch cultivations, we observed a clear effect of Gln consumption on the excretion of ammonia, Ala and Glu, as expected due to their direct connection to Gln metabolism (Doverskog et al., 1997; Zhang et al., 2016). A clear link was also evident between Asp and Glu (see Figure 7D). Both amino acids could be linearly linked to viable PCV; Glu increased constantly, whereas Asp was fleetly consumed. Asn can be converted into Asp and then further into Glu, followed by the building of alpha ketoglutarate. However, after a certain threshold [1.4% (v/v) PCV] presumably caused by a high glutamate concentration, the cells were assumed to be pressured to break down Glu and build alpha ketoglutarate out of pyruvate. Accordingly, Ala was produced and transported out of the mitochondria (see Figures 7F, 8). This behavior has been described in several publications (Altamirano et al., 2001; Sellick et al., 2011; Duarte et al., 2014; Pereira et al., 2018). Most interesting is the fact that this switch happened at an Asp/Glu ratio of ~2 (Figure 7E). We propose that, at this threshold, the cells tend to by-pass the citrate cycle, resulting in less oxygen consumption (see Figure 8). The results indicate that Asp and Glu, in particular, need to be taken into consideration to maintain the respiratory activity and energy metabolism.

The combined technique presented here (capacitance and OUR) will add great value for process characterization and allow the development of control algorithms, especially to maintain respiratory activity. The technique exemplifies a simple tool for metabolic sensing. The metabolic status of the cultured cells can be tracked in real-time. To the best of our knowledge, real-time estimation of a metabolic transition in mammalian cell culture processes has not been reported previously. Future research in this field could include investigations of detailed amino acid fluxes, as well as the dependence of product titer on OURs.

## Conclusion

We have demonstrated that simple bioreactor characterization in terms of *k*_*L*_*a* coefficients and measurement of standard parameters can provide broad information about the cells cultured in this system. Compared to conventional off-gas analyses, the dynamic *k*_*L*_*a* strategy was equally or better suited to calculate OUR trends. Thus, the strategy is highly applicable and easy to implement on multiple scales and in a wide variety of processes, organisms, and cell lines. The generated model allows for real-time visualization of OURs, enabling enhanced understanding of growth characteristics and metabolic reactions with varying process conditions. The presented soft-sensors provide numerous insights: (i) a dynamic *k*_*L*_*a* model needs to be considered in a varying process, (ii) OURs are related more to cell volume than viable cell counts, and (iii) the model cell line switches to another metabolic state when the proportion of Asp to Glu drops in the chosen process setting.

The OUR profile alone gives a first indication of the cellular activity in a process and will add great value to process development. Moreover, a combined soft-sensor with an on-line capacitance measurement presents opportunities for more advanced process optimization through real time monitoring and control of metabolic states.

## Data Availability

All datasets generated for this study are included in the manuscript and/or the Supplementary Files.

## Author Contributions

WS and BS designed the experiments. MP performed the experiments. MP and BS derived the models and analyzed the data. MP and BS wrote the manuscript in consultation with WS and GS.

### Conflict of Interest Statement

MP, BS, and WS are employed by the company Bilfinger Industrietechnik Salzburg GmbH. The remaining author declares that the research was conducted in the absence of any commercial or financial relationships that could be construed as a potential conflict of interest.

## Figures and Tables

**Table:** 

*a*	Specific interfacial area	[m^–1^]
c^*^	Maximum solubility in water at equilibrium	[mol L^–1^]
*c_DO_*	Temperature corrected actual oxygen concentration	[mol L^–1^]
c_L_^*^	Maximum solubility of oxygen in the broth under prevailing gas-phase composition, temperature, and pressure	[mol L^–1^]
c_M_^*^	Maximum oxygen solubility in media	[mol L^–1^]
*c_O2_*	Actual oxygen concentration in the broth	[mol L^–1^]
D	Tank diameter	m
d_i_	Impeller diameter	m
DO	Dissolved oxygen	[%]
*G_in/out_*	Gas flow rate at real conditions in/out of bioreactor	[mL min^–1^]
H	Tank height	m
*k_L_*	Mass transfer coefficient	[m s^–1^]
*k_L_a*	Volumetric oxygen mass transfer coefficient	[s^–1^]
MAPE	Mean absolute percentage error	[%]
N	stirrer speed	[s^–1^ or rpm]
OTR	Oxygen transfer rate	[mol L^–1^ d^–1^]
OUR	Oxygen uptake rate	[mol L^–1^ d^–1^]
*p_O2_*	Partial pressure of oxygen	[Pa]
*Q*	Gas flow rate	[m^3^ s^–1^]
*q_O2_*	Specific oxygen uptake rate	[mol cell^–1^ day^–1^]
*R*	Gas constant	[J mol^–1^ K^–1^]
*T*	Temperature	[°C];[K]
TCC	Total cell concentration	[cells mL^–1^]
τ_P_	Response time	[s]
*v_s_*	Superficial gas velocity	[m s^–1^]
VCC	Viable cell concentration	[cells mL^–1^]
V_L_	Volume of the liquid in vessel	[m^3^]
x_target_	target value	
x_i_	value of sample	
n	number of samples	
X	Biomass concentration	[g L^–1^]
*y_CO2_*	Molar fraction of CO_2_	[%]

## Supplementary Material

Appendix Figure 1 **(A)** Viable cell concentrations (VCC) and **(B)** Packed cell volume (PCV) viable over the time progression of all fed batch runs.

Appendix Figure 2 **(A)** Alanine, **(B)** asparagine, **(C)** aspartate, **(D)** glutamine, and **(E)** glutamate over the time progression of all fed batch runs.

Appendix Figure 3 Algorithm for on-line prediction of metabolic stages in the cells.

## Abbreviations

Ala: Alanine
Asn: Asparagine
Asp: Aspartate
ATP: Adenosine tri phosphate
BAC: Bacterial artificial chromosome
CHO: Chinese hamster ovary
CFD: Computational fluid dynamics
DoE: Design of experiments
FMOC: Fluorenylmethoxycarbonyl
Gln: Glutamine
Glu: Glutamate
HPLC: High performance liquid chromatography
IgG: Immunoglobulin G
mAb: Monoclonal antibody
NADPH: Nicotinamide adenine dinucleotide phosphate
OPA: o-Phthalaldehyde
PA: Process Air
PAT: Process Analytical Technology
PCV: Packed Cell Volume
PID: Proportional Integral Derivative
QbD: Quality by Design
RO: Reverse Osmosis
STR: Stirred Tank Reactor
TCA: Tricarboxylic acid cycle.
