# Supplementary figures and images for: Oxygen Uptake Rate Soft-Sensing via Dynamic kLa Computation: Cell Volume and Metabolic Transition Prediction in Mammalian Bioprocesses
Source: Front Bioeng Biotechnol. 2019 Aug 21;7:195. doi: 10.3389/fbioe.2019.00195 (PMC6712683; doi:10.3389/fbioe.2019.00195)

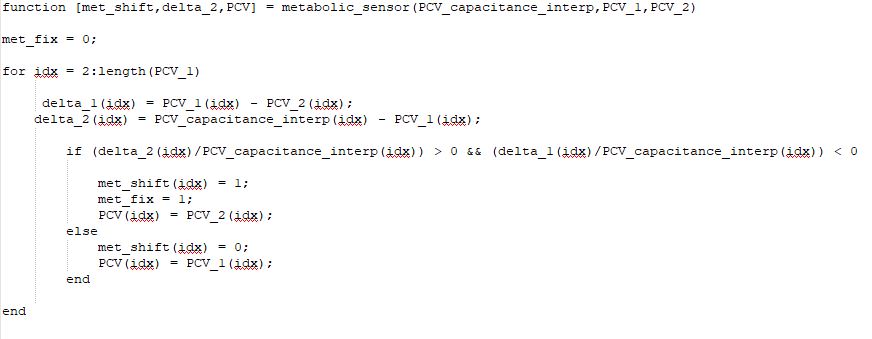

Supplement: Appendix Figure 3 — Algorithm for on-line prediction of metabolic stages in the cells. [file Image_3.JPEG]
